# Food Insecurity among Low-Income Households with Children Participating in a School-Based Fruit and Vegetable Co-Op

**DOI:** 10.3390/children9081250

**Published:** 2022-08-19

**Authors:** Allison N. Marshall, Ru-Jye Chuang, Joanne Chow, Nalini Ranjit, Jayna M. Dave, Mallika Mathur, Christine Markham, Shreela V. Sharma

**Affiliations:** 1 Cizik School of Nursing, The University of Texas Health Science Center at Houston (UTHealth), Houston, TX 77030, USA; 2 Department of Epidemiology, Human Genetics and Environmental Sciences (UTHealth) School of Public Health, The University of Texas Health Science Center at Houston, Houston, TX 77030, USA; 3 Department of Health Promotion and Behavioral Sciences, School of Public Health, University of Texas Health Science Center at Austin (UTHealth), Austin, TX 78701, USA; 4 USDA/ARS Children’s Nutrition Research Center, Baylor College of Medicine, Houston, TX 77030, USA; 5 Department of Health Promotion and Behavioral Sciences, School of Public Health, The University of Texas Health Science Center at Houston (UTHealth), Houston, TX 77030, USA

**Keywords:** dietary behaviors, child health, food security, nutrition, school-based program, food co-op, longitudinal

## Abstract

The purpose of this study was to evaluate the impact of a nutrition intervention on food insecurity among low-income households with children. Data were collected from 371 parent–child dyads in a quasi-experimental evaluation study of a 1-year intervention (*n* = 6 intervention schools receiving Brighter Bites, *n* = 6 wait-list control schools), and longitudinal follow-up of the intervention group 2 years post-intervention in Houston, Texas. Data were collected at three timepoints: at baseline and 1 year for all participants, and at 2 year follow-up for the intervention group (the wait-list control group received the intervention during that time). At baseline, most parents reported food insecurity (60.6%; 70% intervention group, 53.6% control). Food insecurity decreased significantly from 81.3% to 61.7% [(−0.32, −0.07) *p* = 0.002] among intervention participants immediately post-intervention. After adjusting for ethnicity, 2 years post-intervention the predicted percentage of participants reporting food insecurity decreased significantly by roughly 35.4% from 76.4% at baseline to 41.0% [(−0.49, −0.22), *p* < 0.001]. Between-group changes were not significant. The re-sults of this study demonstrated a significant positive impact of Brighter Bites on food security in the short and long-term among low-income households with children, albeit results should be in-terpreted with caution.

## 1. Introduction

Food insecurity is defined as a household-level economic and social condition of limited or uncertain access to adequate food, typically in low-income households [1]. Households with children are more likely to experience food insecurity [2]. In 2019, 13.6% of United States (U.S.) households with children experienced food insecurity, higher than the 9.3% of U.S. households without children who experienced food insecurity [2]. The COVID-19 pandemic resulted in greater hardships for those households who were food insecure or at risk of food insecurity prior to the pandemic resulting in 14.8% of households with children experiencing food insecurity in 2020 [3]. Food insecurity is associated with disrupted eating patterns and reduced food intake [2], as well as lower quality food and poor nutrition [4], all of which are detrimental to psychosocial and physical development and functioning of children.

Food insecurity contributes to household stress which negatively impacts child development, and negatively influences childhood growth and development more directly through nutrition [5]. Links exist between household food insecurity, maternal depression, and child developmental problems, with food insecurity predicting maternal depression and food insecurity and maternal depression independently predicting some child behavior issues [6]. Findings from a review of food insecurity and behavioral and intellectual development of children found that food insecurity negatively impacts a variety of psychosocial, social, behavioral, and academic indicators [7]. A systematic review and meta-analysis supports link between household food insecurity and poor early childhood development among children younger than 5 years, including math and vocabulary skills [8]. Additionally, from US National Health and Nutrition Examination Survey (NHANES) data, six- to 11-year-olds were more likely to have repeated a grade and had lower arithmetic scores when they came from food insufficient households [9].

Disrupted eating patterns among food insecure households and greater difficulties in consistently accessing adequate food because of financial constraints can result in increased consumption of energy-dense, nutrient-deficient foods and decreased consumption of fruits and vegetables [10]. Increased consumption of unhealthy foods and decreased consumption of healthy foods is linked to higher probabilities of various chronic diseases among food insecure individuals [11]. Food insecurity is associated with lower nutrient intakes in children [12]. Results from NHANES data indicate that food insecure children were at risk of lower than recommended intakes of some micronutrients such as vitamin D and magnesium [13]. Food insecurity in childhood has been linked to higher odds of anemia and low height-for-age as compared to children from food secure households [14]; and food insecurity is also associated with poorer general health among children [15].

Due to inadequate nutrition, reduced food intake, or consumption of nutrient poor foods, food insecure individuals may be at higher risk of chronic diseases or chronic disease risk factors such as hypertension, diabetes, and kidney disease [16,17,18]. In addition to the increased risk of chronic diseases, several studies suggest that being food insecure increases the likelihood of being stressed or depressed [19]. Healthy dietary behaviors, including fruit and vegetable consumption, are important for proper growth and development of children and for healthy physical and immune function across age groups [20,21,22]. Therefore, programs to improve food security among families could have far-reaching effects on healthy development and functioning for children, as well as physical and mental health across the lifespan.

The objective of the current study is to evaluate the impacts of a school-based nutrition intervention, the Brighter Bites program, on food insecurity among households participating in the program. Data for this study were derived from an evaluation study that sought to examine the long-term impacts of Brighter Bites programming on dietary intake, parent feeding practices, and home nutrition environment.

## 2. Materials and Methods

### 2.1. Brighter Bites

Brighter Bites is an evidence-based school nutrition intervention for low-income children and their families in the U.S. using a food co-op model where parents in participating schools help bag and distribute fresh fruits and vegetables (F&V) to participating families [23,24]. It is a health promotion program informed by Social Cognitive Theory [25] and Theory of Planned Behavior [26] that increases access to fresh F&V and provides nutrition education targeting increased F&V consumption for participating children and parents. Brighter Bites programming targets attitudes, self-efficacy, and behavioral capability at the individual level, as well as interpersonal-level influences, including communication and organizational-level social norms, nutrition practices, and policies at school; and home environment availability and accessibility of F&V, and provision of F&V at meals and snack-time at home. Programming consists of two 8-week sessions conducted in the Spring and Fall of the school year, consisting of weekly produce distribution at a central location in the school. During produce distributions, participating families each receive roughly 30 pounds of fresh F&V (~50–60 servings) per week, bilingual (Spanish/English) nutrition education via printed booklets, and printed recipe cards distributed weekly in the produce bags, and recipe tastings using produce from that week’s bag that use low-cost and culturally relevant ingredients [24]. Types of fresh F&V included each week vary based on supply and season, with an emphasis on providing culturally relevant, varied fruit and vegetable options. Brighter Bites produce distributions are facilitated by Brighter Bites program staff and supported by parent volunteers. The program targets schools serving low-income populations (>75% of the children participating in the free/reduced price lunch program).

### 2.2. Design

**Figure 1:**
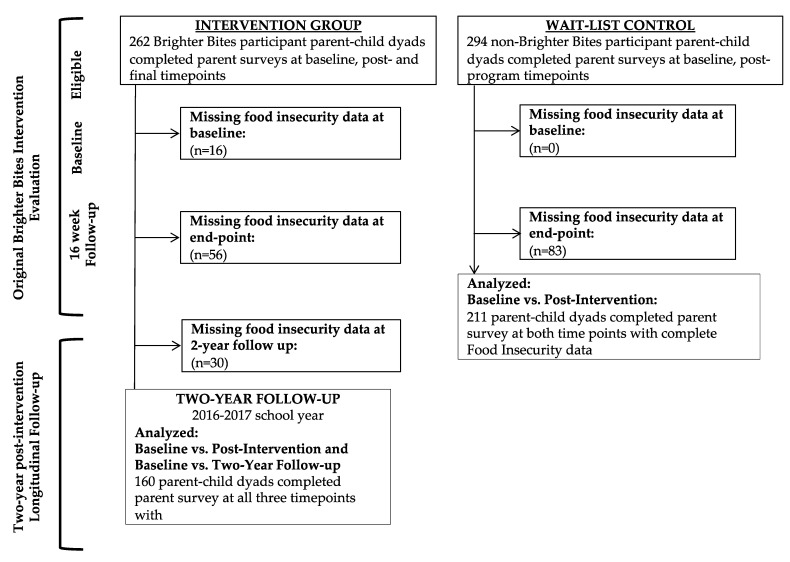
Study flow of analysis of food insecurity from a two-year longitudinal follow-up evaluation of Brighter Bites.

The evaluation study that served as the data source for this study utilized a quasi-experimental non-randomized pre-post design to evaluate the impacts of Brighter Bites programming over a 2-year period compared to a non-equivalent waitlist control group [27]. The study was conducted in two waves in two academic years from 2013–2015 (*n* = 6 intervention schools receiving Brighter Bites and *n* = 6 waitlist control schools), and additional follow-up data were obtained from the intervention group two school years post-intervention (2016–2017) in Houston, Texas [11,12]. The study flow is presented in Figure 1. In the current comparative study, intervention participants received Brighter Bites programming while the comparison group received the Coordinated Approach to Child Health (CATCH) programming, which is an evidence-based obesity-prevention program in schools including classroom, home, and school-wide components to improve healthy eating and physical activity behaviors [28,29]. Because food insecurity was a secondary outcome, it was not included in the power calculation.

School-level eligibility required a student population that included 1st-grade students, and >75% of the student population enrolled in the free/reduced price lunch program. School recruitment consisted of outreach to school administration by program and study staff and an invitation to participate in a study that included Brighter Bites as an opportunity to increase access to F&V among their student population. Subsequent meetings included teaching staff to inform them about the study. Following the school agreement to participate, presentations were made to parents to inform them about the study. Within schools, we recruited 1st-grade classrooms at baseline (2–3 per school). All students in participating classrooms were able to participate in Brighter Bites programming but were only included in analysis if written informed consent was obtained from parents. Parents must be able to read and write English or Spanish to participate. After a full review, this study was approved by the [blinded] Institutional Review Board.

In the current study, we analyzed data from the subsample of the population of 160 parent-child dyads in the intervention group that had food insecurity data at all three time points (baseline, post-intervention, and 2-year follow-up) and 211 control participants for whom food insecurity data were complete at baseline and post-intervention timepoints. Participants were measured at baseline at the start of the school year prior to Brighter Bites programming, and at 9-month follow-up at the end of the second of two 8-week sessions of programming. Subsequently, we obtained 2-year follow-up measures in 2017 only among those in the intervention group since those in the waitlist control group received Brighter Bites post-intervention at the end of the 2015 school year. All intervention and measurement materials were available in Spanish and English. Brighter Bites programming was made available to all students in the participating intervention schools [12], but measurements were only conducted among those who consented. Waitlist control schools implemented the evidence-based CATCH program [28,29] during the study period and received the Brighter Bites programming at the end of 2015, following the 9-month post-intervention measures [23].

### 2.3. Measures

All data were collected via pen-and-paper self-report surveys distributed to and collected from parents through take-home folders and at Brighter Bites distributions. Measures included parent-reported demographics and food security (1 item).

Parent/Caregiver and child demographic data were collected via self-report measures at baseline. We collected demographic data on gender (male or female) and age of child and caregiver, as well as respondent relationship to the child and language spoken at home. We also collected demographic data on caregivers, including race/ethnicity, country of birth, education level, and employment status. Race/ethnicity response options included: Black or African American; Mexican-American, Latino, or Hispanic; White, Caucasian, or Anglo; Asian (Chinese, Indian, Pakistani, Vietnamese, or another Asian country); Native Hawaiian or Other Pacific Islander; American Indian or Alaska Native; more than one race; and other. We also assessed the number of people living in the household and the number of children under age 18 living in the household.

Food security was assessed at all three timepoints (baseline and post-intervention for intervention and control groups, as well as at 2-years for intervention group) using one item: How often in the past 12 months would you say you were worried or stressed about having enough money to buy nutritious food? This item is based on the United States Department of Agriculture (USDA) Household Food Security Survey module [30,31]. Response options included: always, usually, sometimes-about half the time, rarely, and never. Food security responses were dichotomized—we classified respondents answering at least sometimes or more (always, usually, or sometimes-about half the time) as food insecure. We classified respondents as food secure if they responded that they rarely or never were worried or stressed about having enough money to buy nutritious food.

### 2.4. Analysis

We restricted analyses to participants for whom data at all three timepoints were available for those in the intervention group in order to examine the changes immediately post-intervention by comparing between intervention and control group and for changes among intervention participants across all three timepoints. For the control group, data were available only at two timepoints, baseline and post-intervention. Estimates of percent food insecure were calculated using multilevel mixed-effect logistic regression models that adjusted for ethnicity and Brighter Bites attendance to compare the intervention and control group. Estimates of percent food insecure at baseline and at two-year follow-up in the Brighter Bites group only, were calculated using multilevel mixed-effect logistic regression models that adjusted for ethnicity. Descriptive statistics by intervention group status were computed as means and frequency distributions. Sociodemographic differences between the two groups at baseline were tested using independent t-tests or chi-square tests. To account for repeated measures on each subject and cluster-level confounding effects of schools, we estimated the effects of Brighter Bites on food insecurity levels using mixed-effects logistic regressions with subjects and schools as random effects. Changes in food insecurity from baseline to post-intervention within groups and the contrast across groups (delta) were obtained by estimating group-by-time interactions. Additionally, we adjusted for ethnicity for all models and Brighter Bites attendance for models comparing 2-year follow-up and baseline. A *p*-Value of 0.05 was set as the threshold for statistical significance. All analyses were performed using Stata 15.1 (StataCorp, College Station, TX, USA).

## 3. Results

### 3.1. Baseline Demographics

Demographic characteristics of the sample at baseline are presented in Table 1. The mean child age at baseline was 6.14 years (SD 0.36), and the mean parent age at baseline was 35.10 years (SD 7.27). The mean household size for the total sample was 4.93 (±1.74), and the mean number of children younger than 18 years old was 2.63 (±1.18). Most child participants were female (52.2%), most respondents were female (92.5%), and most were Hispanic (77.0%). Most respondents reported speaking English or some English at home (78.8%), and 54.3% of respondents were born in countries other than the United States. There were no significant differences in measured demographics between intervention and control participants aside from ethnicity (*p* = 0.035). At baseline, the majority of parents included in our study sample reported being food insecure (unadjusted prevalence for the full sample was 60.6%; 70% for the intervention group, and 53.6% for the control group).

### 3.2. Estimated Changes in Percent Food Insecure over Time

The post-intervention assessment demonstrated a significant decrease in the prevalence of food insecurity among those in the intervention group. Estimated changes in percent food insecure among parent-child dyads are presented in Table 2. After adjusting for ethnicity and Brighter Bites attendance, from pre to post-intervention the predicted probability of being food insecure decreased significantly by 19.9% from 81.3% to 61.7% [(−0.32, −0.07) *p* = 0.002] among participants receiving Brighter Bites. The control group experienced in food insecurity from 55.3% to 46.2% (9% decrease), albeit not statistically significant [(−0.21, 0.03), *p* = 0.152]. However, the difference in magnitude of change between groups was not statistically significant [−0.11, (−0.28, 0.07) *p* = 0.12]. At 2-year follow-up among those in the intervention group, the decreases in food insecurity persisted; after adjusting for ethnicity, the predicted percentage of participants reporting food insecurity decreased significantly, by roughly 35.4% from 76.4% at baseline to 41.0% 2 years post-intervention [(−0.49, −0.22), *p* < 0.001].

## 4. Discussion

The current study aimed to examine the impact of Brighter Bites, a school-based and theory-grounded nutrition intervention using a co-op model, on food insecurity during a 16-week intervention and over time. At baseline, 60.6% of the study participants reported experiencing food insecurity. Food insecurity is considerably higher compared to the national average of 13.6 percent of the households with children reporting being food insecure [32], albeit expected given that the program targets schools serving low-income households with children. We saw statistically significant within-group decreases in food insecurity among those in the intervention group, but between group changes pre-to-post intervention, while in the direction desired, were not statistically significant. However, the decreases in reported food insecurity were sustained among those in the intervention group after 2 years without continued intervention activities, suggesting that the observed decline in the first year was not due to chance. The results showing decreased food insecurity among Brighter Bites participants presented concur with those of other studies that have demonstrated a positive impact of interventions that improve access to healthy food resulting in reduced food insecurity [33,34]. What is also notable in our study was the sustained decrease in prevalence of food insecurity at 2-year post-intervention follow-up among those in the intervention group. Prior Brighter Bites studies have demonstrated concurrent improvements in consumption of F&V and home nutrition environment among the same cohort of study participants as this study at the 2-year follow-up [23]. These sustained improvements in both food insecurity and healthy eating among those in the Brighter Bites group could likely be due to the combined impact of improving consistent access to fresh produce plus the educational component of Brighter Bites that teaches families grocery shopping, produce storage, and healthy cooking skills which could have a lasting impact on food security.

Although the intervention group experienced significant declines in food insecurity over the 1-year period of the intervention, between-group comparison of changes in food insecurity were not significant. Households in the comparison group also concurrently reported a decrease in the prevalence of food security at 9-month follow-up. This could be due to several reasons, such as the existence of other community efforts or nutrition education programs such as CATCH [28,29] (the program delivered to waitlist control schools) being implemented in the areas of control schools concurrently as the Brighter Bites program that could potentially impact the findings and attenuated the study results. Little is known about the interaction between Brighter Bites and the other healthy eating initiatives. Results of prior studies on assessing the behavioral impact of Brighter Bites demonstrated similar concurrent improvements among control group participants in cooking behaviors, and home nutrition environment [23,24], which could explain our findings. Our results do not allow us to attribute changes in food insecurity to Brighter Bites (which could be due to small sample size and lack of a passive control group), we did see promising sustained impacts of the program that need to be assessed in future fully powered studies with a stringent study design.

Government assistance programs such as Supplemental Nutrition Assistance Program (SNAP), SNAP-Ed (nutrition education component of SNAP), and Women Infants and Children (WIC) programs are proven to be sustainable models to mitigate food insecurity among low-income populations [35,36,37]. Interestingly, while only 30% of Brighter Bites families reportedly participated in government assistance food programs, over 60% were food insecure at baseline. These findings support a need for additional research to understand the factors informing enrollment in government assistance programs and strategies to increase enrollment to be better able to reach all those who qualify for the assistance programs as well as those who need food assistance. Brighter Bites provides, on average, 50 servings of produce on a weekly basis per family; that constitutes roughly 2 additional servings per day for a family of 4. This is not sufficient to meet all the needs regarding produce for a household. Brighter Bites is meant to supplement families with healthy food and facilitate and support behavior change by providing added produce. The results of our study call for more research to understand the structural/systemic issues surrounding food and nutrition insecurity among low-income communities.

### Strengths/Limitations

There are several strengths of this study. This study includes longitudinal measures, including 2-year follow-up. The inclusion of three timepoints of food insecurity measurement for the intervention group is a strength of this study. Additionally, this study uses a control group at 9-month follow-up, as opposed to a single-group pre-post design. Further, many relevant studies have focused on children or adults [38]. This study focused on households with children among low-income populations with important implications for informing nutrition and health promotion programs.

Because this study used a waitlist control, the control group received the programming following the initial intervention period. Thus, no data were available for the control group at the 2-year follow-up measurement time point. However, this study does look at food security over time, which addresses a gap in the literature. Additionally, the inclusion of a control group at baseline and post-intervention still provides valuable insight even without control data at the 2-year follow-up.

One limitation of this study is the reliance on self-reported data. Given that food insecurity, as well as participation in nutrition assistance programs, are sensitive issues, it is possible that social desirability and self-reporting bias may influence these findings. Finally, food security was measured using 1 item, which, to our knowledge, was not validated. While there are other more extensive measurement items for food insecurity, including those used by the USDA [39] and the 2-item Hunger Vital Signs [40], this study used a single item for ease of respondent burden and consistency for comparability across timepoints. Subsequent to this study, Brighter Bites has included the 2-item Hunger Vital Signs to assess the household risk of food insecurity. Results have consistently shown ~70% food insecurity among Brighter Bites participants from 2017–2019, consistent with the baseline findings of this study [41].

## 5. Conclusions

In conclusion, the results of this study demonstrated a significant positive impact of Brighter Bites on food security in the short and long-term among low-income households with children. However, results are to be interpreted with caution since only within-group changes were significant, and the item used to measure food insecurity was not validated. These data support the need for future studies to be conducted to understand the mechanisms and evaluate the impact of programs such as Brighter Bites on food security using validated measures, a rigorous study design, and a study sample powered to this outcome.

## Figures and Tables

**Table 1:** Baseline characteristics of participants.

Characteristics	Total	Intervention Group	Control Group	
	*n* = 371	*n* = 160	*n* = 211	
	**mean (±SD ^4^)**	**mean (±SD ^4^)**	**mean (±SD ^4^)**	***t*-tests** ***p*-Values**
**Child’s age**	6.14 (±0.36)	6.13 (±0.35)	6.14 (±0.36)	0.676
**Parent’s age**	35.10 (±7.27)	35.72 (±7.69)	34.66 (±6.94)	0.182
**Number of people in household**	4.93 (±1.74)	5.02 (±1.98)	4.87 (±1.54)	0.417
**Number of children younger than 18 years old in household**	2.63 (±1.18)	2.62 (±1.06)	2.63 (±1.26)	0.909
	***n* (%)**	***n* (%)**	***n* (%)**	**chi-sq tests** ***p*-values**
**Child’s gender**				
Boy	170 (47.8)	76 (50.3)	94 (45.9)	0.403
Girl	186 (52.2)	75 (49.7)	111 (54.1)	
**Parent’s gender**				
Male	27 (7.5)	13 (8.5)	14 (6.7)	0.517
Female	333 (92.5)	139 (91.5)	194 (93.3)	
**Respondent’s relationship to child**				
Mother	331 (92.2)	142 (92.8)	189 (91.8)	0.710
Father or grandparents	28 (7.8)	11 (7.2)	17 (8.3)	
**Parent’s ethnicity**				
Hispanic	281 (77.0)	125 (80.2)	156 (74.6)	0.035 *
African American	65 (17.8)	29 (18.6)	36 (17.2)	
White	13 (3.6)	1 (0.6)	12 (5.8)	
Other	6 (1.6)	1 (0.6)	5 (2.4)	
**Language spoken at home**				
English or some English	271 (78.8)	105 (75.0)	166 (81.4)	0.156
Spanish only or another language	73 (21.2)	35 (25.0)	38 (18.6)	
**Parent’s country of birth**				
U.S.	155 (45.7)	58 (42.0)	97 (48.3)	0.258
Other countries	184 (54.3)	80 (58.0)	104 (51.7)	
**Parent’s employment status**				
Employed ^1^	180 (53.4)	67 (48.6)	113 (56.8)	0.136
Unemployed ^2^	157 (46.6)	71 (51.4)	86 (43.2)	
**Parent’s highest education level**				
Less than high school graduate ^3^	106 (31.4)	46 (32.9)	60 (30.3)	0.773
High school graduate	91 (26.9)	40 (28.6)	51 (25.8)	
Some college or technical school	86 (25.4)	34 (24.3)	52 (26.3)	
College graduate	55 (16.3)	20 (14.3)	35 (17.7)	

^1^ “Employed” includes those who reported employed for wages, self-employed, and employed in seasonal labor. ^2^ “Unemployed” includes those who reported out of work for more than one year, out of work for less than one year, homemaker, retired, and unable to work. ^3^ “Less than high school graduate” includes those who reported never having attended school or only attended kindergarten, Grade 1 through 8, or Grade 9 through 11. ^4^ SD stands for standard deviation. * Statistical significance at *p* ≤ 0.05.

**Table 2:** Estimated changes in percent food insecure among parent–child dyads (*n* = 371), by intervention condition.

		Intervention (*n* = 160)	Control (*n* = 211)	Net Difference
				**(95% CI ^3^),** ***p*-Value**
**Pre vs. Post (16-weeks), comparison of intervention and control ^1^**				
Percent food insecure				
	Pre-intervention	81.3%	55.3%	
	Post-intervention	61.7%	46.2%	
	Change in percent food insecure over time	−19.9%	−9.0%	−0.11 (−0.28, 0.07)
	*p for change over time*	0.002 *	0.152	*p* = 0.12
**Pre vs. Post (2-year follow-up), intervention only ^2^**				
Percent food insecure				
	Pre-intervention	76.4%		
	Post-intervention	41.1%		
	Change in percent food insecure over time	−35.4%		
	*p for change over time*	<0.001 ***		

Pre vs. post: *n* = 371 parent–child dyads (intervention: control = 160:211); Pre vs. two-year follow-up: *n* = 160 parent–child dyads (intervention only). To account for repeated measures on each subject and cluster-level confounding effects of schools, effects of Brighter Bites on food insecurity levels were estimated using mixed-effects logistic regressions adjusted by subjects and schools as random effects. ^1^ Estimates of percent food insecure were calculated using multilevel mixed-effect logistic regression models that adjusted for ethnicity and Brighter Bites attendance. ^2^ Estimates of percent food insecure at baseline and at two-year follow-up in the Brighter Bites group only, were calculated using multilevel mixed-effect logistic regression models that adjusted for ethnicity. ^3^ CI = confidence intervals. * Statistical significance at *p* ≤ 0.05; *** statistical significance at *p* ≤ 0.001. Note that percentages may not sum to 100 due to rounding of decimals.

## Data Availability

These data are not publicly available.
